# A descriptive analysis of the characteristics and the peer review process of systematic review protocols published in an open peer review journal from 2012 to 2017

**DOI:** 10.1186/s12874-019-0698-8

**Published:** 2019-03-13

**Authors:** Tanja Rombey, Katharina Allers, Tim Mathes, Falk Hoffmann, Dawid Pieper

**Affiliations:** 1 Institute for Research in Operative Medicine, Witten/Herdecke University, Ostmerheimer Str. 200, 51109 Cologne, Germany; 2 Department of Health Services Research, Carl von Ossietzky University Oldenburg, 26111 Oldenburg, Germany

**Keywords:** Peer review, Protocol, Systematic review, Meta-analysis, PROSPERO

## Abstract

**Background:**

An a priori design is essential to reduce the risk of bias in systematic reviews (SRs). To this end, authors can register their SR with PROSPERO, and/or publish a SR protocol in an academic journal. The latter has the advantage that the manuscript for the SR protocol is usually peer-reviewed. However, since authors ought not to begin/continue the SR before their protocol has been accepted for publication, it is crucial that SR protocols are processed in a timely manner.

Our main aim was to descriptively analyse the peer review process of SR protocols published in *‘BMC Systematic Reviews*’ from 2012 to 2017.

**Methods:**

We systematically searched MEDLINE via PubMed for all SR protocols published in *‘BMC Systematic Reviews’* between 2012 and 2017, except for protocols for overviews, scoping reviews or realist reviews. Data were extracted from the SR protocols and Open Peer Review reports. For each round of peer review, two researchers judged the extent of revision (minor/major) based on the reviewer reports. Their content was further investigated by two researchers in a random 10%-sample using PRISMA-P as a guideline. All data were analysed descriptively.

**Results:**

We identified 544 eligible protocols published in *‘BMC Systematic Reviews’* between 2012 and 2017. Of those, 485 (89.2%) also registered the SR in PROSPERO, the majority (87.4%) before first submission of the manuscript for the SR protocol (median 49 days). The absolute number of published SR protocols increased from 2012 to 2017 (21 vs 145 protocols), as did the median processing time (61 vs 142 days from submission to acceptance) and the proportion of protocols requiring a major revision after first peer review (19.1% vs 52.4%). Reviewer comments most frequently addressed the PRISMA-P item *‘Eligibility criteria’*. Overall, 76.0% of the reviewer comments suggested more transparency.

**Conclusions:**

The number of published SR protocols increased over the years, but so did the processing time. In 2017, it took several months from submission to acceptance, which is critical from an author’s perspective. New models of peer review such as post publication peer review for SR protocols should be investigated. This could probably be realized with PROSPERO.

**Electronic supplementary material:**

The online version of this article (10.1186/s12874-019-0698-8) contains supplementary material, which is available to authorized users.

## Background

Systematic reviews (SRs) are conducted to inform clinical practice and decision-making by synthesising and contextualising all relevant evidence that is available regarding a specific research question. Since SRs are typically retrospective in nature [1], it is crucial that their methods, such as the in- and exclusion criteria, outcomes and analyses, are determined a priori, and that deviations from the proposed methods are being reported and justified [2]. Otherwise, methods might be modified post hoc according to the observed results, reflecting an arbitrary, not a systematic approach [3].

One way to establish one’s methods a priori is to develop a SR protocol, that is ‘[ …] a document that presents an explicit scientific ‘road map’ of a planned, uninitiated systematic review’ (p. 3) [4]. If SR protocols are publicly available, they can be compared with the completed SR to assess if deviations from planned methods occurred and whether these biased the results [5]. Some organisations, for example Cochrane [6], the Campbell Collaboration [7], and the Joanna Briggs Institute [8], require that a SR protocol is published before the review is initiated, e.g. in the organisations’ own peer-reviewed journals. Apart from that, it is optional to publish a SR protocol in a peer-reviewed journal. However, in a recent study, we showed that SRs with published protocols tend to be of higher reporting and methodological quality than SRs without published protocols [9].

Authors can also register their SR free of charge with the International Prospective Register of Systematic Reviews, PROSPERO, to establish their methods a priori. To date, over 30,000 SRs are registered in PROSPERO [10]. Registration records are checked against the registry’s eligibility criteria after submission, but there are no further mechanisms of quality assurance (like peer review), leaving the authors responsible for the quality, accuracy and up-to-dateness of their record [11]. Nevertheless, like published protocols, PROSPERO records can be used to assess if deviations from planned methods occurred.

Both, publishing protocols or registering SRs, helps to avoid unnecessary duplication as it allows other researchers to systematically search for them [12, 13]. The advantage of publishing a protocol in an academic journal compared to registration alone is that the manuscript for the SR protocol is usually peer-reviewed before publication. So, in addition to the editor, independent reviewers critically appraise the proposed methods and the protocol’s completeness and transparency. This not only ensures the reporting quality of the SR protocol, but also the methodological quality of the planned SR.

A good peer review takes time. In the context of SR protocols, having a manuscript peer-reviewed means a delay in the generation of new knowledge. This is because it is not advisable for authors to begin or continue conducting their SR before the protocol has been accepted for publication as changes to the planned methods (protocol amendments) may be required. A delay in the generation of new knowledge consequently causes a delay in the dissemination of new knowledge. This is costly for the research community and general public [14] and may even affect the author’s careers [15], hence why it is crucial that SR protocols are processed in a timely manner.

*‘BMC Systematic Reviews’* is an open peer review journal established in 2012. As we have recently shown, it is among the journals publishing the majority of SR protocols [9]. As the journal publishes both, SR protocols and completed SRs with findings, as well as other types of studies related to SRs [16], the peer review statistics published on the journal’s website do not tell us how long the peer review process takes for a SR protocol specifically.

Therefore, the aim of our study was to descriptively analyse the peer review process of SR protocols published in ‘*BMC Systematic Reviews’* from 2012 to 2017. Furthermore, we descriptively analysed the characteristics of these protocols and present trends over time.

## Methods

### Study design

This was a retrospective, observational study based on SR protocols published in *‘BMC Systematic Reviews’* and their Open Peer Review reports, which are all available in open access. There was no a priori study protocol for this study.

### Search strategy

We searched for SR protocols published in *‘BMC Systematic Reviews’* in MEDLINE via PubMed (date of search 8 January 2018) using the following strategy: ‘*protocol AND syst rev [journal]’*.

### Eligibility criteria

We included all SR protocols that were labelled as such and published in *‘BMC Systematic Reviews’* up to and including 2017 regardless of their topic area. Protocols for overviews, scoping- or realist reviews as well as articles that were not SR protocols were excluded.

### Data management and study selection

All records retrieved by the literature search were managed in EndNote (version X8.1, Clarivate Analytics). To accelerate the screening process, we used an approach described by Khangura et al. [17]: One researcher (TR) screened the titles of all records and second researcher (DP) screened only the records excluded by the first researcher. Discrepancies were resolved by discussion.

### Data extraction and data items

Data were extracted from the protocols and Open Peer Review reports (reviewer reports and author comments) into a piloted Excel spreadsheet by one researcher (TR). Where TR was in doubt and in a 10%-sample of all protocols (every tenth protocol), another researcher (KA/TM/DP) double-checked the data. Discrepancies were resolved by discussion. For data items requiring our judgement (e.g. the topic area), we a priori set a cut-off value for the minimum level of inter-rater reliability of 0.8. Items in which the minimum level was not achieved in the 10%-sample were double-checked by another researcher (KA/TM/DP) in every protocol.

The following characteristics were extracted for every protocol: publication year, country of first author’s first affiliation, topic area, whether the authors reported information on competing interests, whether the protocol had already undergone full external peer review as part of an external and non-industry funding process, source of funding (with regard to review), number of authors, date protocol received, number of reviewers, number of revisions, date authors responded (for each revision), date protocol was accepted, date protocol was published and date of PROSPERO registration (if available). The date peer review was completed, level of interest, quality of written English and competing interests were extracted for each reviewer and each revision. We did not investigate any revisions required by the editors.

In addition, we judged the extent of each revision by assessing the reviewer reports in detail. As a rule of thumb, the extent of a revision was classified as *‘major’* if changes to the planned methods (protocol amendments) had to be made which would have prevented to begin or continue the review. It was classified as *‘minor’* if more transparency was required, e.g. by justifying or describing any of the planned methods in greater detail. These judgements were made independently by two researchers (TR, TM/DP) for every protocol.

In a randomly selected 10%-sample of the included protocols, one researcher (TR, KA) allocated every methods-related comment the PRISMA-P item (no. 8 to 17) that best described it [5]. For each item, we also captured whether a reviewer’s comment suggested a protocol amendment or more transparency. By assessing the reports of author comments, we then checked whether the authors had implemented the proposed changes or not.

### Statistical analysis

All data were descriptively analysed. For continuous data, we calculated means and standard deviations (SDs), medians and its interquartile ranges (IQRs). For categorical data, we calculated frequencies and proportions.

We used the dates a protocol was first submitted, peer-reviewed (for each reviewer), the authors responded (for each revision), accepted and published to calculate the following durations: Submission to first peer review, submission to acceptance, submission to publication, acceptance to publication.

All statistical analyses were performed using SAS for Windows, version 9.4 (SAS Institute Inc., Cary, NC).

## Results

### Search results

Our literature search resulted in 693 records, of which 544 (78.5%) were SR protocols that fulfilled the inclusion criteria of our study. The remaining records were either not a protocol or were protocols for primary studies, overviews, scoping- or realist reviews.

### Basic characteristics of the included protocols

Almost half of the 544 protocols (*n* = 269) were published in 2016 or 2017, and most first authors were affiliated with an institution from Canada or the UK (in total 58.3%). Nearly half the protocols (44.1%) were for therapeutic reviews. There was a competing interest statement in every protocol (100%). Ten protocols (1.8%) had already undergone full external peer review, which means they only underwent editorial peer review as per the journal policy [18]. In almost two thirds of the protocols (62.9%) the source of funding for the review was a non-profit institution. The median number of authors was 6. The majority of all protocols (89.2%) were also registered in PROSPERO, 87.4% of those before submission of the protocol (Table 1).

**Table 1:** Basic characteristics of the included protocols

Protocol characteristics	*n* = 544
Publication year	
2012	3.9%
2013	10.3%
2014	17.0%
2015	19.5%
2016	22.8%
2017	26.7%
Country (most common)	
Canada	32.2%
UK	26.1%
Other	22.6%
Australia	7.4%
Germany	7.0%
USA	4.8%
Topic area of SR	
Therapy (treatment, prevention)	44.1%
Other (i.e. methodology)	27.0%
Epidemiology (i.e. prevalence)	22.6%
Diagnosis	4.2%
Prognosis	2.0%
Source of funding of the SR	
Non-profit	62.9%
For-profit/mixed	2.4%
No funding	18.6%
Not reported/unclear	16.2%
Number of authors	
Mean (SD)	6.5 (3.6)
Median (IQR)	6 (4–8)
Registered in PROSPERO (*n* = 485; 89.2%)	
Before submission / acceptance	87.4%
After submission / acceptance	2.5%
Same day	10.1%

Abbreviations: *SR* Systematic review; *SD* Standard deviation; *IQR* Interquartile range

### Peer review characteristics and processing time

Overall, there were 1.3 ± 0.7 reviewers and 1.1 ± 0.6 revisions per protocol. For 50.0% of all protocols, we classified the extent of revision after the first round of peer-review as *‘minor’*, for 37.9% as *‘major’*. In the remaining protocols, there was no (open) peer-review (7.2%), no revision was required by the reviewer(s) (4.0%), or the reviewer report was missing (0.9%). After the second round of peer review, 9.3% of the 162 protocols that were not accepted after the first revision required a major revision. 50.6% required a minor revision and 40.1% did not require a further revision. Ten protocols were peer-reviewed three times or more often. 

In 6.4% of the protocols, at least one reviewer indicated that the protocol was of limited interest. The quality of written English was found to need some language corrections or not suitable by at least one reviewer in 30.4% (Additional file 1).

The median processing time from submission to first peer review, from submission to acceptance, from submission to publication and from acceptance to publication was 36, 98, 113 and 14 days, respectively. Processing times were longer in protocols requiring a major revision after first peer review compared to protocols requiring a minor revision (see Table 2).

**Table 2:** Extent of revision after first round of peer-review and processing times (in days)

Extent of revision	major (*n* = 206)	minor (*n* = 272)
Submission to first peer-review		
Days, mean (SD)	52 (44)	48 (48)
Days, median (IQR)	40 (25–60)	33 (21–54)
Submission to publication		
Days, mean (SD)	150 (69)	119 (65)
Days, median (IQR)	134 (103–177)	105 (77–143)
Submission to acceptance		
Days, mean (SD)	134 (69)	104 (65)
Days, median (IQR)	120 (87–157)	87 (60–124)
Acceptance to publication		
Days, mean (SD)	17 (12)	15 (9)
Days, median (IQR)	13 (9–20)	14 (9–18)

In the remaining protocols (*n* = 66) there was no reviewer, no revision or no comments from the reviewer entailing a revision

### Trends over time

There was a steady increase in the number of protocols published in *‘BMC Systematic Reviews’* over the years. The proportion of protocols whose corresponding SR was registered in PROSPERO increased from 2012 to 2017 (80.1 to 91.7%), with a peak in 2016 with 96% (Table 3). Of those published between 2015 and 2017, 90–94% were registered prior to submission of the protocol. Between 2012 and 2014 it was 65–77%. The median time from registration in PROSPERO to protocol submission was much longer in 2017 compared to 2012 (62 vs 22 days, respectively) in SRs registered prior to protocol submission (*n* = 424).

**Table 3:** Time trends in basic and peer-review characteristics and processing times

Year	2012	2013	2014	2015	2016	2017	Overall
	(*n* = 21)	(*n* = 56)	(*n* = 92)	(*n* = 106)	(*n* = 124)	(*n* = 145)	(*n* = 544)
Number of protocols registered in PROSPERO, Absolute (%)	17 (80.1%)	42 (75.0%)	81 (88.0%)	93 (87.7%)	119 (96.0%)	133 (91.7%)	485 (89.2%)
Registered prior submission							
Absolute (%)	11 (64.7%)	30 (71.4%)	62 (76.5%)	84 (90.3%)	112 (94.1%)	125 (94.0%)	424 (87.4%)
Days, Mean (SD)	45 (45)	67 (77)	78 (110)	87 (131)	101 (107)	110 (129)	94 (117)
Days, Median (IQR)	22 (7–85)	43 (13–71)	36 (11–93)	29 (8–114)	71 (28–127)	62 (22–152)	49 (15–120)
Submission to first peer-review							
Days, Mean (SD)	28 (13)	35 (24)	32 (16)	35 (20)	55 (55)	75 (65)	50 (48)
Days, Median (IQR)	29 (19–42)	32 (22–41)	29 (21–41)	32 (20–46)	39 (23–70)	52 (31–95)	36 (22–57)
Submission to acceptance							
Days, Mean (SD)	74 (42)	77 (44)	82 (55)	91 (44)	109 (52)	165 (85)	111 (69)
Days, Median (IQR)	61 (46–105)	77 (51–95)	75 (46–105)	80 (56–117)	103 (72–138)	142 (103–214)	98 (64–139)
Submission to publication							
Days, Mean (SD)	91 (43)	101 (46)	99 (55)	110 (47)	124 (54)	180 (87)	128 (70)
Days, Median (IQR)	86 (59–127)	99 (73–118)	91 (64–120)	101 (74–136)	118 (84–148)	158 (117–222)	113 (82–155)
Acceptance to publication							
Days, Mean (SD)	17 (21)	24 (32)	18 (10)	18 (12)	14 (7)	16 (11)	17 (15)
Days, Median (IQR)	12 (0–22)	15 (11–20)	16 (11–21)	16 (11–21)	13 (9–17)	12 (9–19)	14 (9–19)
Number of reviewers							
Mean (SD)	0.9 (0.6)	1.1 (0.5)	1.1 (0.6)	1.4 (0.8)	1.1 (0.6)	1.7 (0.8)	1.3 (0.7)
Median (IQR)	1 (1–1)	1 (1–1)	1 (1–1)	1 (1–2)	1 (1–1)	2 (1–2)	1 (1–2)
Number of revisions							
Mean (SD)	0.9 (0.8)	1.0 (0.5)	1.2 (0.7)	1.2 (0.6)	1.0 (0.4)	1.2 (0.5)	1.1 (0.6)
Median (IQR)	1 (0–1)	1 (1–1)	1 (1–2)	1 (1–2)	1 (1–1)	1 (1–1)	1 (1–1)
Extent of first revision, Absolute (%)							
Major	4 (19.1%)	14 (25.0%)	25 (27.2%)	38 (35.9%)	49 (39.5%)	76 (52.4%)	206 (37.9%)
Minor	11 (52.4%)	34 (60.7%)	52 (56.5%)	55 (51.9%)	63 (50.8%)	57 (39.3%)	272 (50.0%)
No revision required	1 (4.8%)	3 (5.4%)	6 (6.5%)	2 (1.9%)	3 (2.4%)	7 (4.8%)	22 (4.0%)
No (open) peer review	5 (23.8%)	4 (7.1%)	8 (8.7%)	10 (9.4%)	8 (6.5%)	4 (2.8%)	40 (7.2%)
Reviewer report missing	0 (0%)	1 (1.8%)	1 (1.1%)	1 (0.9%)	1 (0.8%)	1 (0.7%)	4 (0.9%)

Abbreviations: *SD* Standard deviation; *IQR* Interquartile range

The mean number of reviewers almost doubled between 2012 and 2017 (0.9 and 1.7, respectively) although it did not increase continuously over time. However, there was a large difference between 2016 and 2017 (increase from 1.1 to 1.7). There were no apparent trends in the proportion of protocols which did not require a revision, nor in the proportion of protocols that require one revision, two revisions and three or more revisions (Additional file 2). The proportion of protocols requiring a major revision as per our judgement increased steadily over the years (from 19.0% in 2012 to 52.4%), but the largest difference occurred between 2016 and 2017 (from 39.5 to 52.4%). The proportion needing language corrections increased from 23.1 to 40.7% between 2012 and 2017 (Additional file 1).

Median processing times also increased over time. In the years 2012 to 2015, it took about one month from submission to first peer review (values ranged from 29 to 32 days), while in 2017 it was 52 days. The median duration from submission to acceptance was 2.5 times higher in 2017 (142 days) than in 2012 (61 days) and the median duration from submission to publication of 86 days in 2012 almost doubled to 158 days in 2017. For the time between acceptance and publication, there was no consistent trend over the years (ranging from 12 to 16 days).

For comparison, the time from submission to acceptance across all manuscripts submitted to *‘BMC Systematic Reviews’* in 2017 was 168 days and 16 days from acceptance to publication [19].

### Content of reviewer comments

From our random 10%-sample, we had to exclude one protocol for which no reviewer comments were available, resulting in 53 protocols for further analyses based on PRISMA-P. Overall, there were 450 comments (on average 8.5 ± 9.0 per protocol), 342 of which suggested more transparency (76.0%) and 108 which suggested a protocol amendment (24.0%). Suggestions for more transparency were implemented in 85.1% and protocol amendments in 73.7%; non-implementation was justified by the authors for all but two comments, both which suggested a protocol amendment.

More than half of the protocols received comments regarding PRISMA-P item 8 *‘Eligibility criteria’* (66.0%; with an average of 2.1 ± 2.7 comments per protocol) and item 9 *‘Information source’* (52.8%). With 13.2% each, comments on *‘Data management’, Criteria for quantitative synthesis’* and *‘Confidence in cumulative evidence’* were least common (Table 4).

**Table 4:** Content of reviewers’ comments (including each reviewer and revision)

Content of reviewers’ comments		Proportion of protocols with comments (*n* = 53)	Comments per protocol (mean ± SD) (*n* = 53)	Proportion of comments suggesting amendments (*n* = 450)
PRISMA-P item				
8	Eligibility criteria	66.0%	2.1 ± 2.7	16.1%
9	Information sources	52.8%	0.9 ± 1.1	39.6%
10	Search strategy	34.0%	0.6 ± 1.2	50.0%
11a	Data management	13.2%	0.1 ± 0.3	0.0%
11b	Selection process	30.2%	0.5 ± 0.9	12.5%
11c	Data collection process	24.5%	0.3 ± 0.7	11.1%
12	Data items	34.0%	0.5 ± 0.9	31.0%
13	Outcomes and priorization	20.8%	0.4 ± 0.9	21.1%
14	Risk of bias in individual studies	49.1%	0.7 ± 0.9	31.6%
15a	Criteria for quantitative synthesis	13.2%	0.2 ± 0.5	11.1%
15b	Aspects of quantitative synthesis	41.5%	0.9 ± 1.5	14.0%
15c	Additional analyses	39.6%	0.7 ± 1.4	31.4%
15d	Type of summary if quantitative synthesis not appropriate	15.1%	0.2 ± 0.6	16.7%
16	Meta-bias (es)	17.0%	0.2 ± 0.4	10.0%
17	Confidence in cumulative evidence	13.2%	0.1 ± 0.3	42.9%

The proportion of comments suggesting protocol amendments was highest for PRISMA-P item 10 *‘Search strategy’*, where 50.0% of all comments suggested protocol amendments, followed by item 17 *‘Confidence in cumulative evidence’* (42.9%) and item 9 *‘Information sources’* (39.6%). They were implemented in 56.3, 100 and 93.8% of the time, respectively.

## Discussion

Our study shows that the number of protocols published in *‘BMC Systematic Reviews’* has increased tremendously since the journal’s launch, from *n* = 21 in 2012 to *n* = 145 in 2017 (overall *n* = 544). The proportion of protocols requiring a major revision was 37.9% and has also increased with time. Three quarters of reviewer comments suggested more transparency. Most protocols were also registered with PROSPERO (89.2%), an increasing proportion before the manuscript had been submitted (reaching 94.0% in 2017).

While the time from acceptance to publication was constantly about two weeks over the years, the time from submission to publication almost doubled and went from just under three months in 2012 to over five months in 2017. This is how long it takes for a primary study from first submission to acceptance [20]. Since it takes about 1.3 years on average to conduct and publish an entire SR on medical interventions [21], the time it takes to publish a SR protocol seems to be disproportionately high.

From an author’s perspective, long processing times might have far-reaching implications. Although authors should wait until their protocol is accepted for publication, they might choose to continue working on their SR in the meanwhile. This holds the risk that valuable comments from the peer review might not lead to changes in the SR’s methodology, which might be critical unless the part requiring a revision has not been commenced or finished by then. For example, a revision of the eligibility criteria might result in a new search strategy.

Waiting for the protocol to be accepted causes a delay in the conduct of the SR and consequently in the dissemination of new knowledge [14]. Moreover, it is likely to complicate budget and project planning for authors of SRs for several reasons. People who have worked on the protocol might change or the project may simply run out of money due to the long time that publication of the protocol takes. This in turn might entail that the corresponding SR will never be published [22]. In another study, we found that about one-third of SRs remains unpublished 3–5 years after the protocol has been available [9], thus it remains unclear whether submitted but unpublished protocols exist.

The most likely reason for the increased duration from submission to publication is the increase in the proportion of protocols that required major revisions. As we have shown, protocols requiring a major revision after the first round of peer review took about a month longer (median 29 days) to get published than those requiring a minor revision. But why did more protocols require a major revision?

One explanation might be that the quality of initially submitted manuscripts for SR protocols became slightly worse over time, perhaps due to a change in the authors who submit SR protocols. One can assume that publishing protocols has become more popular over the years, while in the beginning only groups which are experienced in performing SRs submitted protocols. This is supported by the fact that the geographical scope in PROSPERO [10], as well as in our study (Additional file 2) has changed with time. Other aspects, such as the number of reviewers/revisions or the time from acceptance to publication, did not follow a clear trend over time in our study, although we found a large increase in the number of reviewers between 2016 and 2017. This suggests that the peer review policy may have been changed during recent years.

Another explanation why more and more protocols required a major revision might be the introduction of PRISMA-P in 2015. Nowadays, it is required by *‘BMC Systematic Reviews’* that the authors follow the checklist and submit it as an additional file [18]. Otherwise their submission will be returned as incomplete, which of course delays the peer review process. Furthermore, peer reviewers may have become more critical as it is likely that the 17-item checklist goes beyond what they had previously looked for. This would also explain why most reviewer comments suggest more transparency.

Because the time from submission to first peer review has also steadily increased from 2015 onwards, another explanation might be the increasing difficulties in finding appropriate peer reviewers given the increase in scientific publications in general [23]. However, it is important to note that there is not only a high burden on peer reviewers, but also on all editors (in-chief, associate, handling) [24].

About 9 out of 10 protocols were also registered in PROSPERO. It is known, that SRs with published protocols are more often registered with PROSPERO than SRs without [9]. However, an interesting finding was that the majority (87.4%) of SRs was registered with PROSPERO before the protocol had been submitted. Paradoxically, PROSPERO urges caution not to register too early and that the review protocol should be complete before submitting the registration request [25]. But this is just the case after peer review is completed. PRISMA-P recommends that, if the SR is registered in a publicly accessible registry, the name of the registry and registration number should be included in the protocol. Although these statements are not contradicting, they do not provide clear instructions for authors of SR protocols.

One major argument for publishing a protocol of a SR is to receive input regarding the review scope and review methodology from independent peer reviewers, who themselves are experienced researchers. However, in a recent study we found that SRs with published protocols had older searches compared to SRs without a published protocol; in 52.2% of the SRs, the final search had already been performed before submitting the protocol for publication [9]. This fits to the finding of this study, that authors only implemented protocol amendments regarding the search strategy in 56.3%, while overall protocol amendments were implemented in 73.7%.

Naturally, authors play an important role in the peer review process, too. We found that most of the reviewer’s comments on methodological issues suggested more transparency, not amendments to the protocol. The former could be avoided to some extent if authors paid more attention to this matter in the preparation of their manuscript, and described and justified their methods more carefully in the initial submission.

One could argue that for protocols, which did not require any amendments, the registration in PROSPERO would have been sufficient. That is because all potential advantages of publishing SR protocols, apart from peer review, could also be achieved through registering the SR with PROSPERO (provided that information is presented in the same amount of detail as they would have been in a protocol). Furthermore, a recent study found that registered reviews were of higher quality than non-registered reviews [26]. However, in practice it would turn out to be very difficult to identify protocols that will benefit most from a peer review a priori. In a random sample of 300 reviews published in 2014, only 4% mentioned that the SR has been registered [27]. However, another study found that, in a random sample of 150 SRs published in 2015, 19% all reviews actually were registered [28].

An advantage of PROSPERO is that the registration and status of the SR can be easily updated and details or the reference for the published review can be added. If the SR has never been completed, another possibility is to switch its status to *‘abandoned’* including details on reasons for that, although this is seldom done [29]. Since any previous versions of an entry are kept, amendments can be made transparent. This is not feasible with published protocols and authors therefore need to report all protocol amendments in the actual review. Despite these useful features, we feel that PROSPERO could be further optimised; the structure of PROSPERO records should be aligned with the PRIMSA-P checklist to facilitate registration for authors of SR protocols.

### Strengths and limitations

This was a study of all SR protocols published in *‘BMC Systematic Reviews’* since it was launched in 2012 up to and including 2017. Despite the large number of analysed protocols (*n* = 544), the journal only served an example. However, it is the open peer review journal, in which most SR protocols are published (39.7%), followed by BMJ Open (23.4%) [9]. It is also important to notice that our data only come from published protocols and the associated reviewer reports and author comments. Thus, it remains unclear how protocols have been dealt with that did not get published.

Although most endpoints of this study were based on data like dates and frequencies, we had to make judgements regarding some endpoints. To be as objective as possible, we did not check back with any peer reviewers, protocol authors or the editors, which means that our judgements might vary from the original ones. To increase internal validity, the reviewer reports for each protocol were assessed by two researchers independently regarding their extent of revision, though.

We used PRISMA-P as a simplified method of content analysis and we might have misclassified some comments. Moreover, the 10%-sample of protocols might be too small for meaningful analysis. However, we had the opportunity to include a total of 450 reviewer comments. Lastly, we did not analyse whether changes in PROSPERO were made after the protocol had been finally approved. Status updates are not often done in PROSPERO [9], though, so their extent should be small.

## Conclusions

The increasing number of published protocols can be interpreted as a desirable trend, but the long and steadily increasing processing time is not acceptable from an author’s perspective. When protocols are submitted, a timely and well-performing peer review system is needed. It has been stressed that due to the increasing number of scientific articles the workload imposed on individual reviewers appears to be reaching a `breaking point’; for protocols of SRs it has already been reached [24]. New models of peer review for SR protocols, such as post publication peer review [30], should be investigated. This could probably be realized with PROSPERO. What authors of SR protocols can do now, is to pay special attention to be transparent when describing and justifying the planned methods.

## Additional files

Additional file 1: Time trends in judgement of level of interest and quality of written English. (PDF 16 kb)

Additional file 2: Time trends in basic and peer-review characteristics and processing times – additional data. (PDF 19 kb)

## Abbreviations

BMC: BioMed Central
BMJ: British Medical Journal
IQR: Interquartile range
OR: Odds Ratio
PRISMA-P: Preferred Reporting Items for Systematic Review and Meta-Analysis
SAS: Statistical Analysis System
SD: Standard deviation
SR: Systematic review
