# Supplementary material for: A descriptive analysis of the characteristics and the peer review process of systematic review protocols published in an open peer review journal from 2012 to 2017
Source: BMC Med Res Methodol. 2019 Mar 13;19:57. doi: 10.1186/s12874-019-0698-8 (PMC6415341; doi:10.1186/s12874-019-0698-8)
Supplement: Supplementary file 1 — Time trends in judgement of level of interest and quality of written English. (PDF 16 kb) [file 12874_2019_698_MOESM1_ESM.pdf]

Additional file 1: Time trends in judgement of level of interest and quality of written English.

Year	2012	2013	2014	2015	2016	2017	Overall
Level of interest assessed*	n = 13	n = 23	n = 38	n = 66	n = 111	n = 140	n = 391
Judged to be limited	0.0%	13.0%	5.3%	7.6%	6.3%	5.7%	6.4%
Quality of written English assessed**	n = 13	n = 23	n = 39	n = 71	n = 112	n = 140	n = 398
Judged to be not suitable/ in need of some language corrections	23.1%	21.7%	23.1%	25.4%	25.9%	40.7%	30.4%

*In the remaining n = 153 there was no reviewer, the reviewer report was missing or the level of interest has not been reported.

** In the remaining n = 146 there was no reviewer, the reviewer report was missing or the quality of written English t has not been reported.
