# Supplementary material for: A descriptive analysis of the characteristics and the peer review process of systematic review protocols published in an open peer review journal from 2012 to 2017
Source: BMC Med Res Methodol. 2019 Mar 13;19:57. doi: 10.1186/s12874-019-0698-8 (PMC6415341; doi:10.1186/s12874-019-0698-8)
Supplement: Supplementary file 2 — Time trends in basic and peer-review characteristics and processing times – additional data. (PDF 19 kb) [file 12874_2019_698_MOESM2_ESM.pdf]

Additional file 2: Time trends in basic and peer-review characteristics and processing times – additional data.

**Year**	**2012 (n = 21)**	**2013 (n = 56)**	**2014 (n = 92)**	**2015 (n = 106)**	**2016 (n = 124)**	**2017 (n = 145)**	**Overall (n = 544)**
Country*							
Canada	52.4%	42.9%	39.1%	23.4%	23.4%	29.7%	32.2%
UK	23.8%	19.6%	22.8%	33.1%	33.1%	25.5%	26.1%
Other	14.3%	21.4%	17.4%	24.2%	24.2%	31.0%**	22.6%
Australia	9.5%	3.6%	5.4%	6.5%	6.5%	6.2%	7.4%
Germany	0.0%	8.9%	7.6%	7.3%	7.3%	3.5%	7.0%
USA	0.0%	3.6%	7.6%	5.7%	5.7%	4.1%	4.8%
Number of revisions							
0	28.6%	10.7%	10.9%	9.4%	8.9%	4.1%	9.0%
1	57.1%	75.0%	63.0%	64.2%	82.3%	75.9%	72.1%
2	9.5%	14.3%	21.7%	25.5%	8.9%	17.9%	17.3%
≥ 3	4.8%	0.0%	4.4%	0.9%	0.0%	2.1%	1.7%
Number of reviewers							
0	23.8%	7.1%	8.7%	9.4%	6.5%	2.8%	7.2%
1	66.7%	80.4%	70.7%	48.1%	80.7%	40.7%	61.4%
2	9.5%	10.7%	19.6%	34.0%	9.7%	40.7%	24.4%
≥ 3	0.0%	1.8%	1.1%	8.5%	3.2%	15.9%	7.0%

*of first author’s first affiliation **Of those: Brazil 15.6%, Sweden 13.3%, Iran 8.9%, South Africa 6.7%, Others 55.6%.
